# Liquid biopsy–based epigenetic signatures for early detection of prostate cancer: a systematic review

**DOI:** 10.3389/fonc.2026.1800705

**Published:** 2026-05-08

**Authors:** Wisdom Deborah Cleanclay, Irrinus Fonyuy Kintung, Fabrice Banadzem Kernyuy, Nina Ghislaine Yensii, Boluwatife Oluwafemi Fagbohun, Emmanuel Chimuebuka Amadi, Niels Nguedia Kaze

**Affiliations:** 1 Department of Biochemistry, College of Science and Technology, Covenant University, Ota, Nigeria; 2 Covenant Applied Informatics and Communication Africa Centre of Excellence (CApIC–ACE), Covenant University, Ota, Nigeria; 3 Covenant University Public Health and Wellbeing Research Cluster (CUPHWERC), Covenant University, Ota, Ogun State, Nigeria; 4 Afrique One Reach, Centre Suisse de Recherche Scientifique, Abidjan, Côte d'Ivoire

**Keywords:** prostate cancer, liquid biopsy, DNA methylation, microRNA, epigenetic biomarkers, early detection, non-invasive diagnostics, biomarker panels

## Abstract

**Introduction:**

Prostate cancer (PCa) is one of the most common cancers among men globally. Early detection remains challenging due to the limited specificity of prostate-specific antigen (PSA) testing, which is widely used for screening and diagnosis. Emerging evidence highlights the critical role of epigenetic alterations, particularly DNA methylation and non-coding RNAs, including microRNAs and long non-coding RNAs, in PCa initiation and progression. Notably, these molecular signatures can be detected in body fluids such as blood and urine, thereby offering promising opportunities for the development of non-invasive diagnostic strategies.

**Methods:**

This systematic review was conducted in accordance with PRISMA 2020 guidelines (PROSPERO ID: CRD420251062598). PubMed, Web of Science, Cochrane Library, and Google Scholar were searched for studies published between 2015 and 2025 that evaluated epigenetic signatures in liquid biopsies for PCa detection. Eligible studies included case–control, cohort, cross-sectional, and diagnostic accuracy designs, and data on study characteristics, biomarker types, detection methods, and diagnostic performance were independently extracted.

**Results:**

Sixty-seven studies met the inclusion criteria from 5,364 screened records. These comprised 14 DNA methylation studies, 51 miRNA studies, and 2 studies with combined epigenetic signatures. Epigenetic biomarkers demonstrated consistent diagnostic potential across multiple liquid biopsy matrices, including serum, plasma, urine, and seminal fluid. GSTP1 and RASSF1A were the most frequently reported DNA methylation markers and showed moderate performance as single markers; however, multi-gene methylation panels achieved higher accuracy. In contrast, microRNAs, particularly when combined into panels, frequently exceeded PSA, especially within the diagnostic grey zone (4–10 ng/mL). miR–21, miR–141, and miR–375 were the most consistently dysregulated miRNAs, although study heterogeneity remained substantial. Overall, studies integrating methylation and miRNA markers with PSA demonstrated improved sensitivity and specificity.

**Conclusion:**

Epigenetic biomarkers detected in liquid biopsies show strong potential as non-invasive tools for early prostate cancer diagnosis. Combining DNA methylation, miRNA markers, and PSA may improve diagnostic accuracy compared with PSA alone. Future studies should prioritise standardised methods, cross-platform validation, and large multicentre cohorts.

**Systematic review registration:**

https://www.crd.york.ac.uk/prospero/, identifier CRD420251062598.

## 1 Introduction

Prostate cancer (PCa) is one of the most significant global health challenges, being the second most commonly diagnosed male cancer and a leading cause of cancer–related deaths among men (1). Despite advances in screening and treatment, early detection remains a major clinical challenge. Prostate–specific antigen (PSA) testing, though sensitive, lacks specificity, leading to false positives, missed diagnoses, and an increase in cases of unnecessary biopsies (2). Moreover, tissue biopsy, the confirmatory diagnostic standard for PCa, is invasive, costly, and associated with discomfort and may lead to urinary tract complications (3). In addition, PCa is a biological and clinically heterogeneous disease, with diverse molecular subtypes and aggressive clinical outcomes across populations. This heterogeneity is influenced by genetic, epigenetic, metabolic, inflammatory, and microenvironmental factors, as well as both androgen–dependent and non–androgen receptor signalling pathways (4). This highlights the urgent need for novel, non–invasive, and reliable biomarkers that can correctly distinguish malignant from benign conditions and stratify clinically significant tumours (5).

In this context, epigenetic dysregulation, referring to heritable changes in gene expression occurring without alterations in the DNA sequence, has emerged as a hallmark of PCa initiation and progression. Key epigenetic mechanisms such as DNA methylation, histone modifications, and regulation by non–coding RNA, all of which modulate chromatin architecture and transcriptional activity (6, 7). Among these, aberrant DNA methylation is among the most extensively characterised and represents one of the earliest molecular events in prostate tumorigenesis. Frequently, observed hypermethylation of regulatory or tumour suppressor genes such as *GSTP1*, *APC*, *RASSF1A*, and *RARβ2* leads to their transcriptional silencing, thereby promoting malignant transformation (8). Conversely, global and gene promoter hypomethylation, observed in elements such as *LINE–1*, *PLAU*, and *HPSE*, contributes to chromosomal instability and metastatic potential. However, hypomethylation is a less commonly reported mechanism in PCa (9). These methylation signatures are detectable in liquid biopsy specimens (blood, urine, saliva, and semen), rendering them highly attractive for non–invasive diagnostics and disease monitoring.

Similarly, noncoding RNAs, particularly microRNAs (miRNAs) and long non–coding RNAs (lncRNAs), are another important level of epigenetic control in PCa (10). These molecules modulate mRNA stability, chromatin organisation, and transcriptional regulation, playing pivotal roles in tumour initiation, progression, metastasis, and therapeutic resistance. Several miRNAs, including *miR–21*, *miR–125b*, *miR–141*, *miR–221*, and *miR–375*, have been shown to function as oncogenes or tumour suppressors, depending on their targets and cellular context (10–12). Furthermore, their dysregulated expression profiles correlate with disease stage, Gleason score, and biochemical recurrence, supporting their potential as non–invasive biomarkers detectable in plasma, serum, urine and saliva (13).

Another epigenetic mechanism of prostate tumorigenesis is histone modification. Acetylation, methylation, phosphorylation, and ubiquitination of histones regulate nucleosome dynamics and transcriptional accessibility. These influences key cancer–related pathways such as androgen receptor signalling, DNA damage repair, and epithelial–mesenchymal transition (14). Also, alterations in histone–modifying enzymes like histone deacetylases, histone methyltransferases, and demethylases have been implicated in the aggressiveness, metastatic potential, and therapy resistance of PCa (15). Although histone modifications, including acetylation and methylation, have been detected in circulating nucleosomes, their translational application remains limited by technical challenges in sample preparation, quantification, and cross-platform reproducibility (13).

In addition, extracellular vesicles (EVs), particularly exosomes, have emerged as important components of liquid biopsy in cancer. They carry a diverse cargo of biomolecules, including microRNAs, DNA, proteins, and other subcellular components that reflect the molecular state of their cells of origin (16). As such, EVs serve as stable carriers of tumour-derived epigenetic signals in circulation, enhancing the detectability of biomarkers in body fluids. Dogra et al. (17) noted the “dark matter” of EVs, referring to previously underexplored molecular components with potential diagnostic and prognostic value. This positions EVs as a critical link between tumour biology and non-invasive biomarker detection.

Furthermore, advances in analytical platforms, including quantitative reverse transcription polymerase chain reaction (RT–qPCR) and next–generation sequencing enables sensitive detection and quantification of circulating miRNAs and DNA methylation markers, facilitating clinical translation. Collectively, epigenetic signatures integrate genetic predisposition with environmental and metabolic influences. Their stability, reversibility, and detectability in liquid biopsies (blood, urine, and seminal plasma) make them promising candidates for early, non-invasive diagnosis and longitudinal disease monitoring (18).

**Figure 1:**
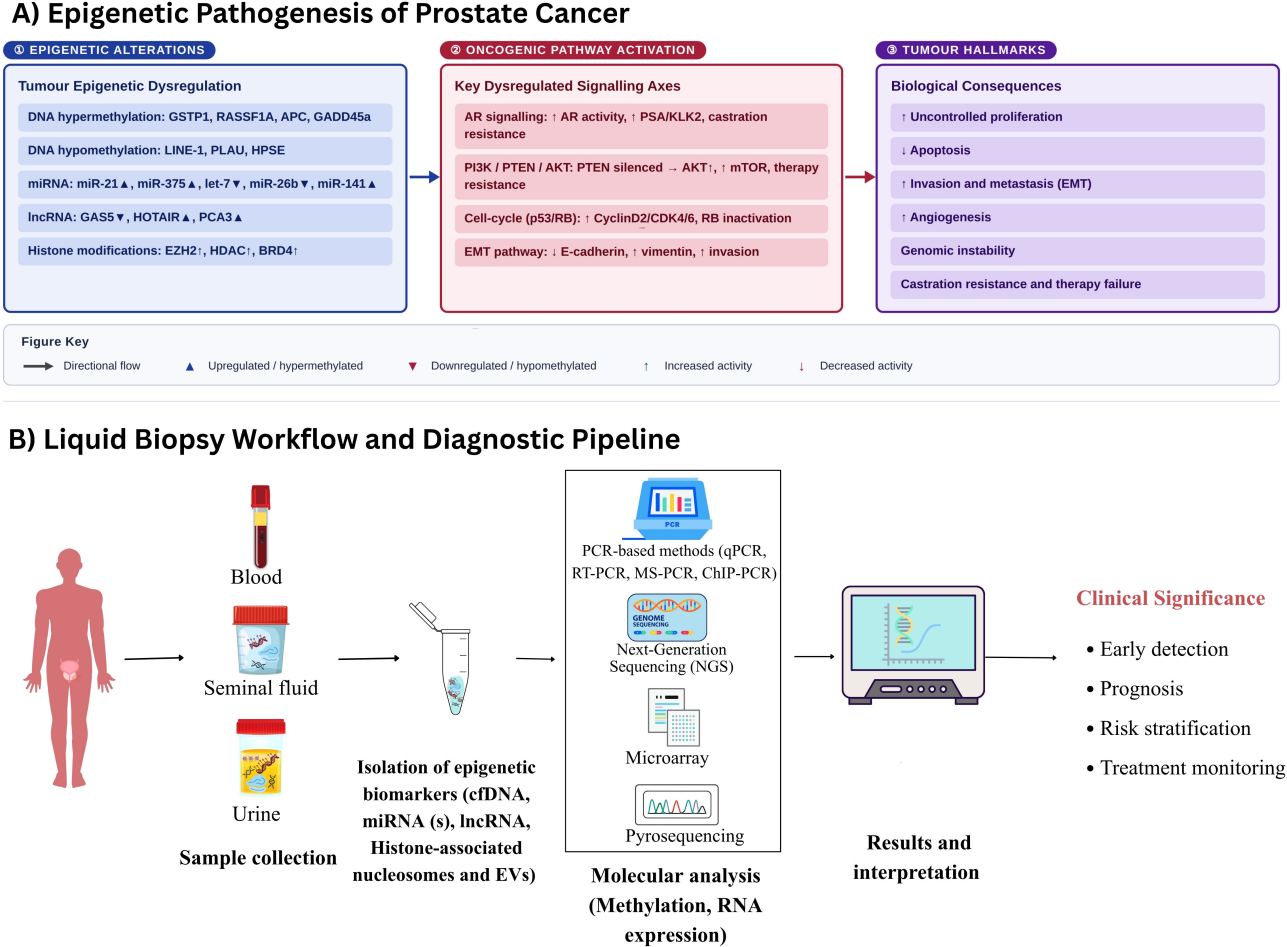
Epigenetic landscape and liquid biopsy pipeline in prostate cancer. **(A)** Illustrates how epigenetic alterations (DNA hypermethylation of GSTP1/RASSF1A, miRNA/lncRNA dysregulation) drive oncogenic pathways (AR and PI3K/AKT/mTOR signalling), resulting in tumour hallmarks like EMT, metastasis, and castration resistance. **(B)** Displays the translational pipeline where biomarkers (methylated cfDNA, ncRNAs, histones, EVs) are extracted from blood, seminal fluid, and urine. These are analysed via PCR, NGS, and Microarrays for clinical applications in early detection, prognosis, and treatment monitoring. Adapted from (91).

This review presents evidence on key epigenetic biomarkers, including DNA methylation, histone modifications, and noncoding RNA expression. Specifically, it aims to: (i) Assess the diagnostic accuracy, reproducibility, and methodological robustness of these biomarkers across biological matrices and analytical platforms; (ii) Find existing limitations, potential biases, and knowledge gaps that impede clinical translation; (iii) Explore integrative and translational opportunities for incorporating validated epigenetic assays into precision diagnostic frameworks. Ultimately, this review seeks to advance the development of standardised, clinically applicable, and minimally invasive epigenetic biomarkers to improve prostate cancer detection, patient stratification, and management (**Figure 1**).

## 2 Methodology

### 2.1 Protocol and registration

This systematic review was conducted in accordance with the PRISMA 2020 guidelines. The review protocol was prospectively developed and registered with PROSPERO (Registration ID: CRD420251062598). This research is a systematic review of published data and did not involve the direct participation of human subjects or animals. Hence, approval from an institutional review board or ethics committee was not required.

### 2.2 Literature search strategy

An online search was conducted to identify relevant studies evaluating the diagnostic utility of epigenetic biomarkers in prostate cancer. The databases searched included PubMed, Web of Science, Cochrane Library, and Google Scholar. Searches were conducted using a combination of Medical Subject Headings (MeSH) and free–text terms. The main search term “prostate” or the MeSH term “Prostatic Neoplasms” was combined with epigenetic–related terms encompassing DNA methylation (“methylation,” “hypermethylation,” “hypomethylation,” “DNA Methylation”), general epigenetic mechanisms (“epigenetic,” “epigenetics,” “Epigenesis, Genetic”), non–coding RNAs including microRNAs (“microRNA,” “miRNA,” “miR*,” “MicroRNAs”) and long non–coding RNAs (“lncRNA,” “long non–coding RNA,” “non–coding RNA,” “Long Noncoding RNA”), as well as histone–related processes (“histone,” “histone modification,” “Histones”). Boolean operators (AND/OR) and truncations were applied to refine and maximise search sensitivity. The search was restricted to studies published between January 2015 and June 2025. No language restrictions were applied, and studies published in non–English languages were considered for inclusion.

### 2.3 Inclusion and exclusion criteria

The PICOS guideline was used to establish selection criteria, comprising Population, Intervention, Comparators, Outcomes, and Study Design. Eligible studies were selected based on alignment with the PICOS framework and on investigating epigenetic biomarkers for the non–invasive diagnosis or early detection of prostate cancer. Only original clinical research that involved human participants was considered. To maintain transparency and methodological clarity, no variations from the PICOS framework were applied. The eligibility criteria were defined as follows:

- Population: Studies including men at risk of, suspected to have, or with histologically confirmed PCa and male participants with benign conditions or healthy controls were eligible as comparator groups.
- Interventions: Studies investigating DNA methylation, miRNAs, lncRNAs, or histone modifications for PCa diagnosis or early detection in liquid biopsies. Both single and multi–gene biomarker panels were eligible.
- Comparators: Standard diagnostic tools (PSA, DRE, histopathology/biopsy) and non–cancer control groups (healthy men, benign prostate disease).
- Outcomes: Diagnostic accuracy (sensitivity, specificity, AUC), early detection performance, association with disease aggressiveness.
- Study designs: Original clinical studies including case–control, cross–sectional, cohort, and diagnostic accuracy studies (randomised or non–randomised).

Exclusion criteria:

- Studies on non–human subjects, animal, or cell–line only studies.
- Reviews, commentaries, editorials, or conference abstracts with incomplete data.
- Studies not featuring any epigenetic signature or reporting diagnostic outcomes.
- Studies without comparator groups.

### 2.4 Study selection

All retrieved records were imported into Rayyan for systematic management. All duplicates identified were verified, manually cross–checked, and removed before screening commenced. Screening was conducted in two stages. Title and abstract screening were conducted by two reviewers, who independently evaluated titles and abstracts against the predefined eligibility criteria. Discrepancies were resolved through discussion, with arbitration by a third reviewer when necessary. Full–text articles of eligible studies were retrieved and independently assessed by the same two reviewers. Reasons for exclusion at this stage were documented in a screening log. While systematic reviews were excluded from the synthesis, their reference lists and those included articles were screened to identify additional relevant primary studies.

### 2.5 Data extraction

The data extraction process was carried out independently by three reviewers (IFK, KFB and BOF) using a well–designed form in Microsoft Excel 2024. Extracted data covered study characteristics (first author, year of publication, country, study design, and sample size), participant features (age, patient characteristics, and type of controls), biomarker information (epigenetic marker(s) studied, assay method, and liquid biopsy source such as semen, saliva, urine, or blood), and reported outcomes (diagnostic accuracy parameters such as sensitivity, specificity, and AUC). To reduce bias, both reviewers cross–checked their data and reconciled any differences through discussion and consensus. Upon persistence of disagreements, a senior researcher, WCD, reviewed the data extraction process and provided the final decision.

## 3 Results

### 3.1 Screening results

**Figure 2:**
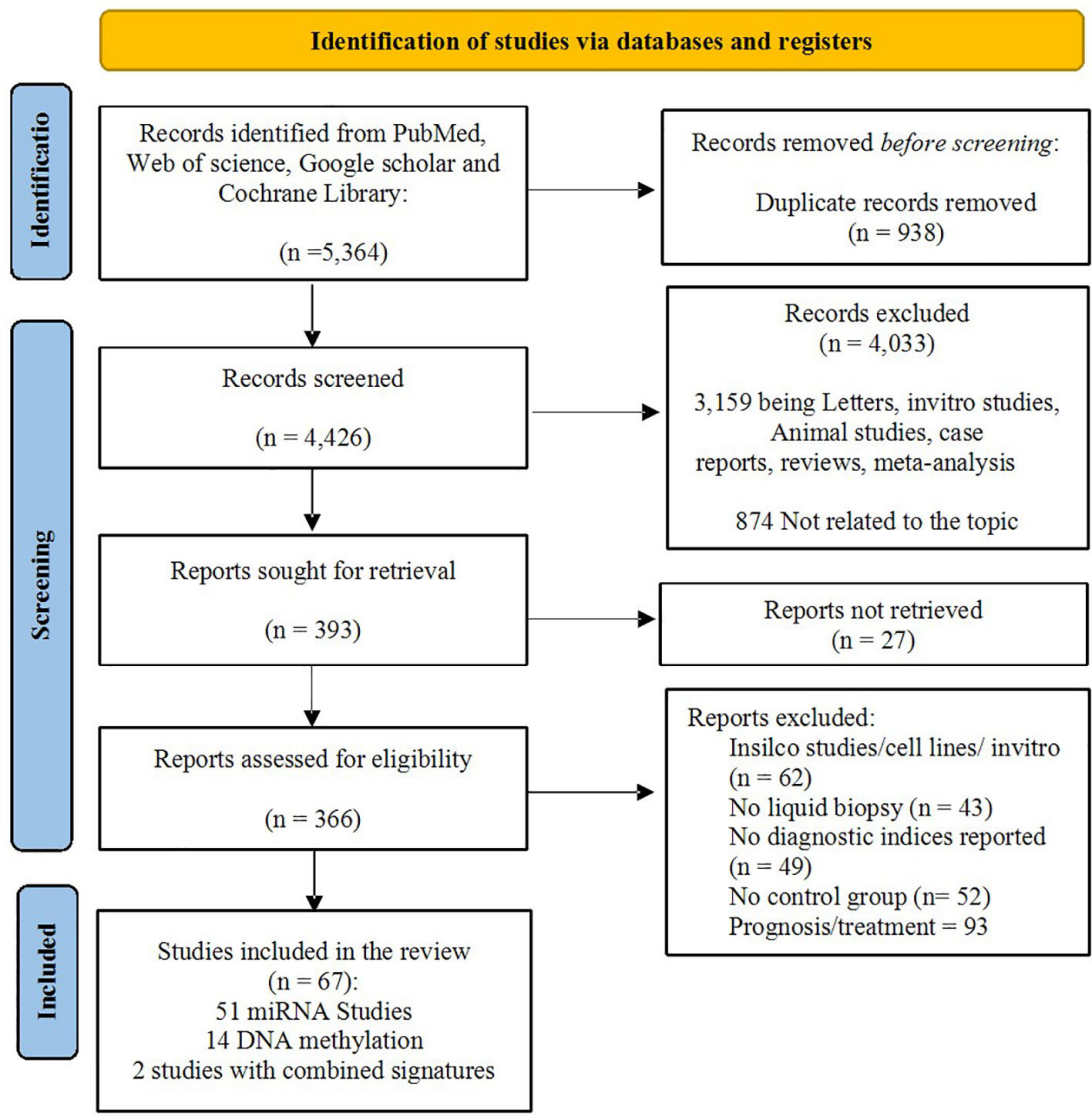
PRISMA flow diagram showing study selection process.

The initial database search retrieved 5,364 records from PubMed, Web of Science, Google Scholar, and the Cochrane Library. Out of these records, 938 duplicates were removed, and 4,426 unique records were screened by title and abstract. Of these, 4,033 were excluded; 3,159 were reviews, case reports, letters, or *in vitro* and animal studies, while the remaining were unrelated to the research question (n = 874). Full texts were sought for 393 articles, with 27 unobtainable despite repeated attempts. The 366 eligible full–texts were reviewed in detail, of which 290 were excluded. The most frequent reasons for exclusion were studies conducted in silico or *in vitro* (62 studies), lack of relevance to liquid biopsy (43 studies), and studies lacking diagnostic performance (49 studies). Additional exclusions were due to the absence of an appropriate control group (52 studies) and studies that focused solely on prognosis or treatment outcomes rather than diagnostic accuracy (94 studies). Ultimately, 67 studies fulfilled all eligibility criteria and were included in the review, comprising 14 studies on DNA methylation, 51 microRNA biomarker studies, one study involving both miRNA and DNA methylation and one study involving both miRNA and lncRNA. The complete study selection process is presented in the PRISMA flow diagram (**Figure 2**). Full details of all included studies are provided in the **Supplementary Material**.

### 3.2 Distribution of studies by year

**Figure 3:**
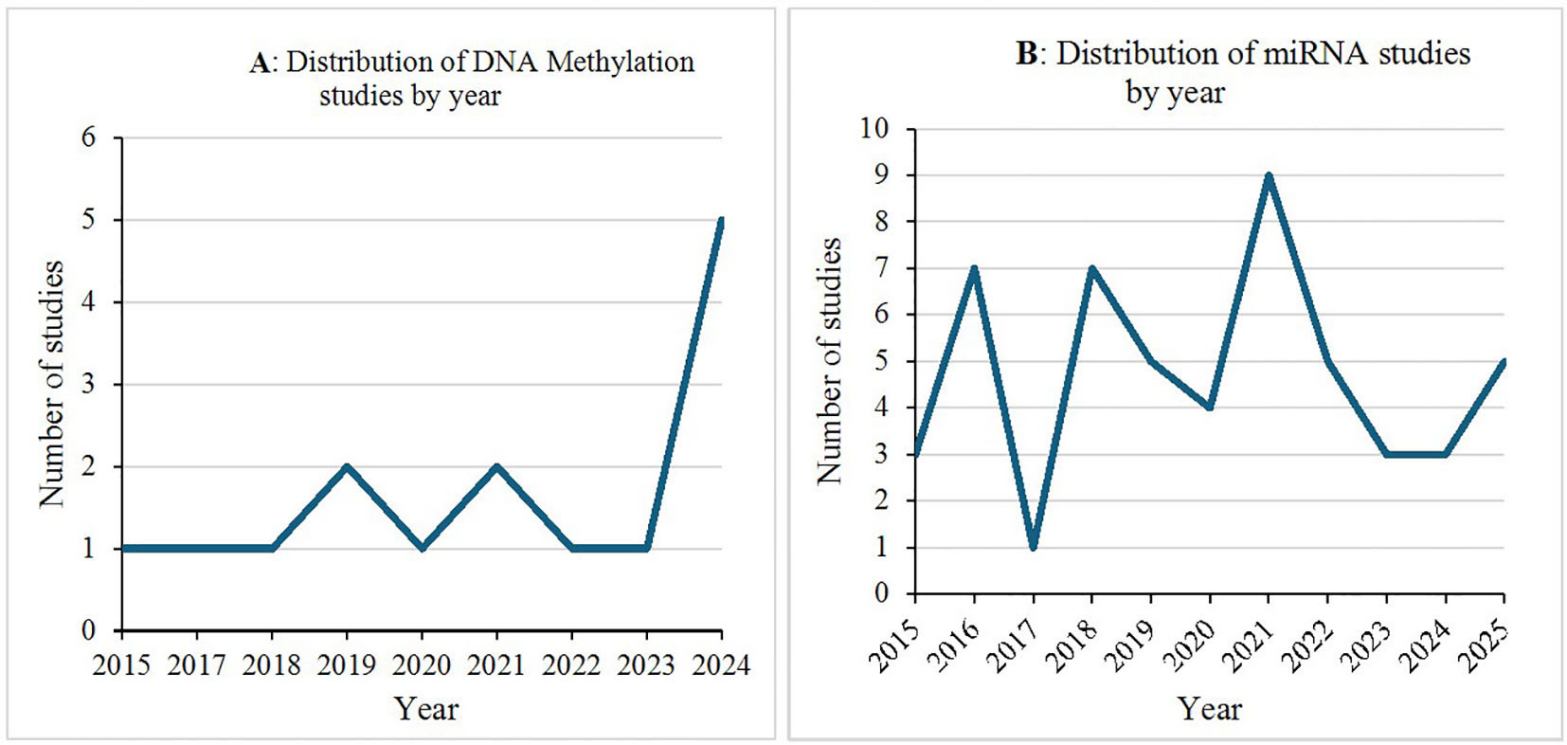
Distribution of selected studies by year; **(A)** Distribution of DNA Methylation studies by year, **(B)** Distribution of miRNA studies by year.

Among the included studies, there was an observable increase in the number of publications investigating epigenetic signatures as diagnostic biomarkers for PCa over time. Studies on DNA methylation showed a gradual rise, peaking in 2024 with 5 studies, while no eligible studies were identified for 2025 (**Figure 3A**). In contrast, studies focusing on miRNAs peaked in 2021 (9 studies), followed by a decline in subsequent years, although a modest resurgence was observed in 2025 with 5 included studies (**Figure 3B**). Notably, only two studies explored integrated multi-modal epigenetic signatures: one combining miRNA and DNA methylation (19), and another combining miRNA and lncRNA (20), published in 2018 and 2025, respectively. Collectively, these trends emphasise the expanding role of epigenetic biomarkers in PCa diagnosis and the emerging importance of integrative, multi-modal approaches.

### 3.3 Risk of bias and applicability assessment (QUADAS–2)

**Figure 4:**
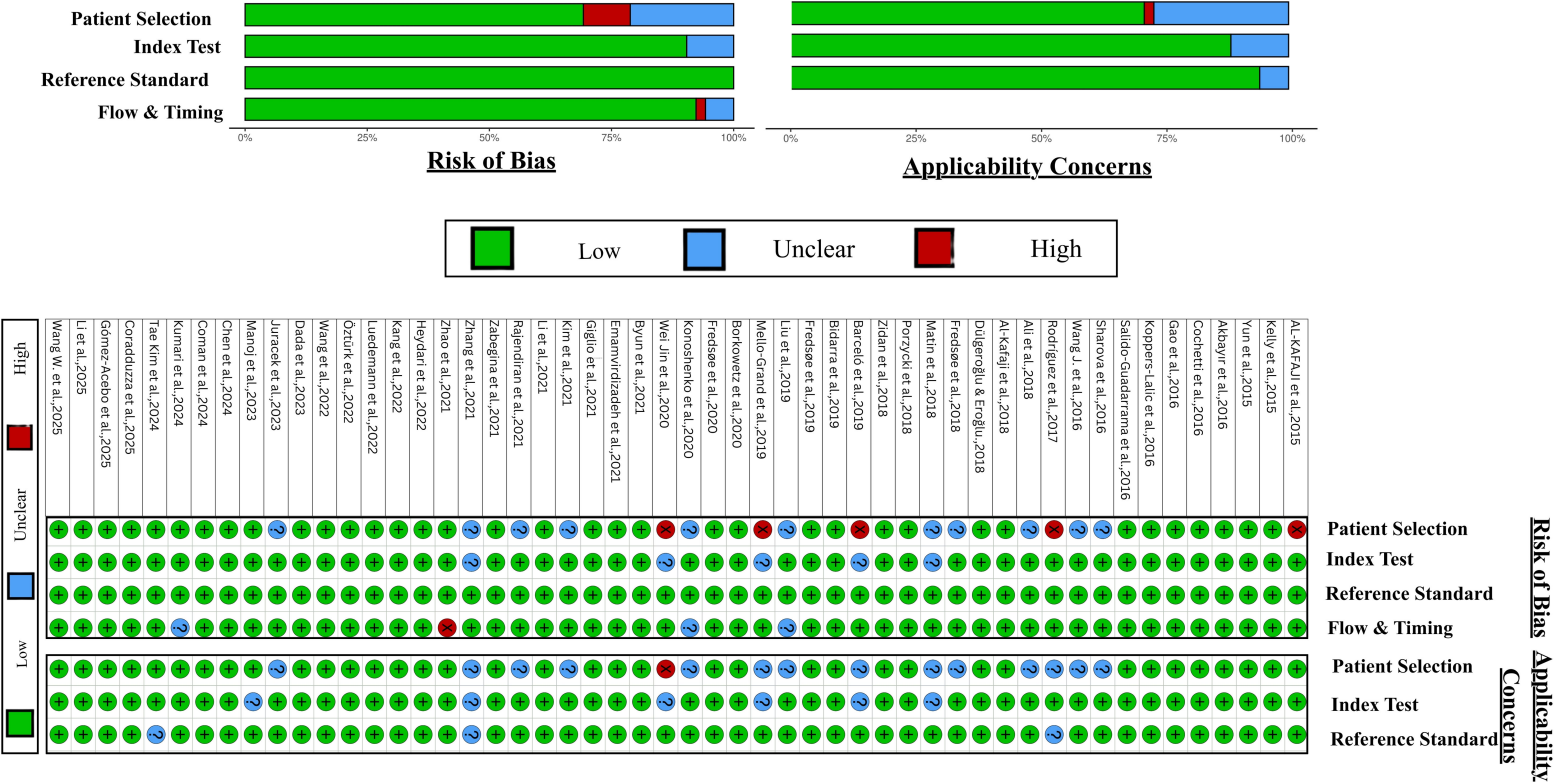
Risk of bias and applicability assessment for miRNA studies, using QUADAS 2.

**Figure 5:**
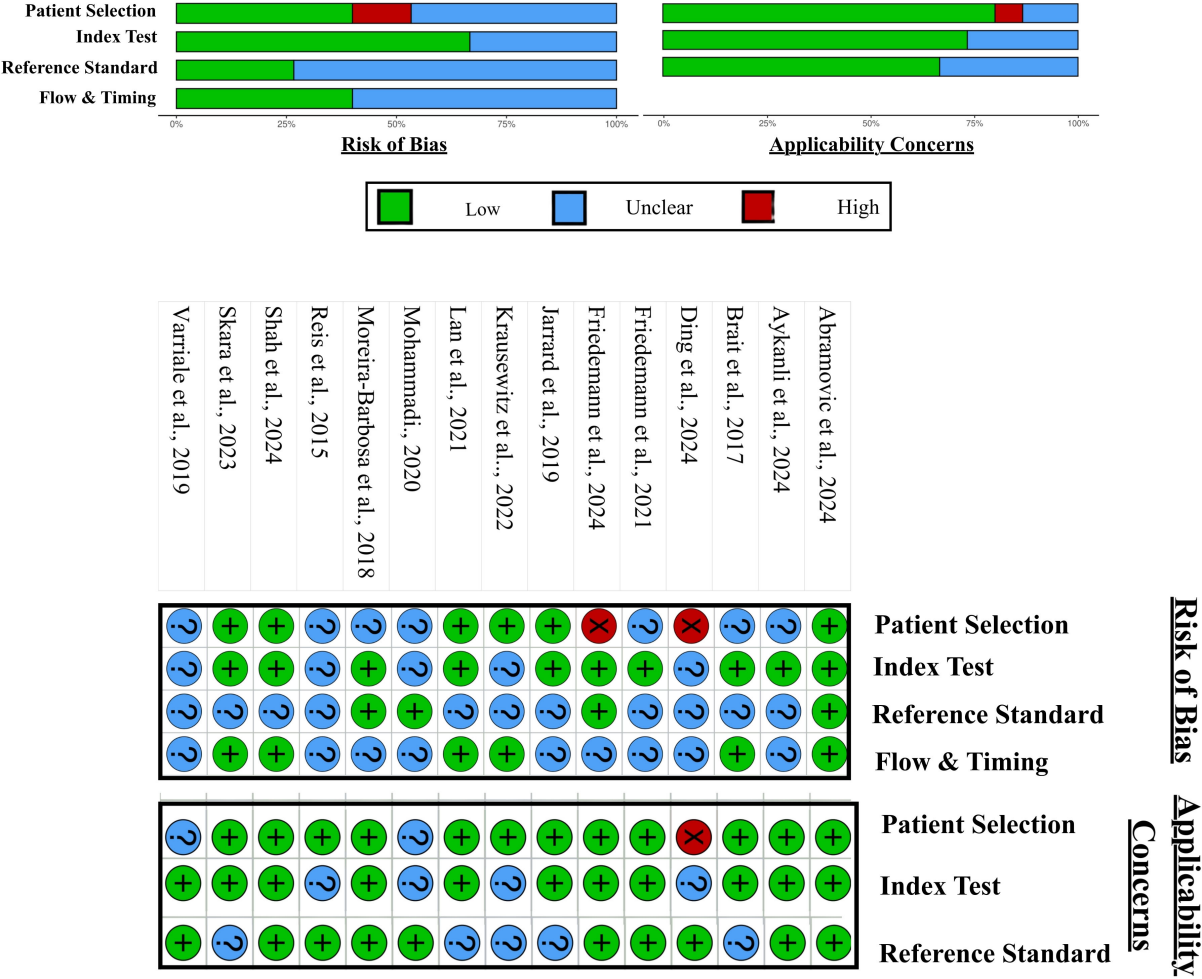
Risk of bias and applicability assessment for DNA methylation studies, using QUADAS 2 tool.

Risk of bias was evaluated using the QUADAS–2 tool across four domains: patient selection, index test, reference standard, and flow/timing. Overall, the studies demonstrated moderate methodological quality, with most rated as low or unclear risk of bias. The patient selection and flow/timing domains contributed most to potential bias, largely due to non–consecutive sampling, lack of blinding, retrospective designs, and incomplete reporting of participant flow or time intervals between index tests and reference standards. In contrast, the index test and reference standard domains generally showed low to moderate risk, reflecting adequate use of histopathological confirmation and validated molecular assays in most studies. Applicability concerns were generally low; however, variations in sample type, assay methods, and population diversity may affect comparability across studies. Risk of Bias and Applicability assessment of miRNA and DNA methylation studies is summarised in **Figure 4** and **Figure 5** respectively.

### 3.4 Study characteristics

All included studies exclusively employed non–invasive liquid biopsy specimens, including serum, plasma, urine, saliva, and seminal plasma, with no tissue–based assays reported. The majority of the studies used serum samples for both DNA methylation and miRNA analysis. Case–control designs were the most used, while a few studies utilised prospective cohorts, and one was a blinded clinical trial. Study populations were predominantly from Asia (China, Japan, Korea), followed by Europe (Italy, Germany, Spain), with limited representation from Africa (two studies from Egypt and one from South Africa). Notably, race or ethnicity was not explicitly reported in most studies, thereby limiting the evaluation of biomarker variability across populations. Sample sizes ranged from 20 to over 500 participants, typically including treatment–naïve, biopsy–confirmed prostate cancer patients. Control groups comprised benign prostatic hyperplasia, healthy individuals, and, in some studies, precancerous lesions.

A summary of study characteristics stratified by biomarker category is provided in **Table 1**. In addition, individual study details are presented in stratified sub–tables: **Table 2** (DNA methylation biomarkers by sample type), **Table 3** (miRNA biomarkers by sample type), and **Table 4** (Comparative diagnostic performance of PSA, DNA methylation, and miRNA biomarkers in prostate cancer detection by sample type).

**Table 1:** Summary of study characteristics by epigenetic biomarker category.

Feature	DNA Methylation studies (n=14)	miRNA studies (n = 51)	Combined epigenetic studies (n = 2)
Study designs	Case–control (predominant); 1 prospective observational	Case–control (predominant); 1 blinded clinical trial; prospective cohorts	Case–control
Liquid biopsy matrices used	Serum, plasma, urine, seminal plasma	Serum, plasma, urine, whole blood, saliva, semen	Urine; plasma
Sample size range (PCa/Control)	13–87 PCa; 4–155 controls	6–425 PCa; 3–407 controls	74–87 PCa; 15–32 controls
Control groups used	BPH, healthy males, ASAP, HGPIN	BPH, healthy males, prostatitis, precancerous lesions	BPH, asymptomatic controls, morphologically normal tissue
Dominant assay method	MS–qPCR, ddPCR, pyrosequencing	RT–qPCR (validation); NGS, microarray (discovery)	MS–qPCR; RT–qPCR
Comparator	PSA, tPSA, fPSA, histopathology	PSA, histopathology, Gleason score	PSA, histopathology

BPH, Benign prostatic hyperplasia; ASAP, Atypical small acinar proliferation; HGPIN, High–grade prostatic intraepithelial neoplasia; MS–qPCR, Methylation–specific quantitative PCR; ddPCR, Droplet digital PCR; RT–qPCR, Reverse transcription quantitative PCR; NGS, Next–generation sequencing; PSA, Prostate–specific antigen; tPSA, Total PSA; fPSA, Free PSA.

**Table 2:** DNA methylation biomarkers in liquid biopsy, stratified by sample type.

Sample	Gene(s)	Assay method	Study (Country)	Key diagnostic outcomes
Serum	RASSF1A, GSTP1	OBBPA–ddPCR	Friedemann et al., 2021 (21) (Germany)	RASSF1A AUC = 0.64–0.80; GSTP1 AUC = 0.51–0.55 vs BPH; fPSA AUC = 0.81–0.86
	GSTP1, HDAC, DNMT3A, DNMT3B	Real–time PCR	Mohammadi et al., 2020 (22) (Iran)	HDAC: AUC=0.84, SN=95%, SP=65.25%; GSTP1: AUC=0.78
	DACT–2	MS–qPCR	Lan et al., 2021 (23) (China)	DACT–2 methylation rate=0.745, SN=81.8%, SP=75.0%
	MCAM, ERα, ERβ	MS–qPCR	Brait et al., 2017 (24) (USA)	MCAM: AUC=0.66, SN=65.5%, SP=73.3%
	GADD45a	Pyrosequencing	Reis et al., 2015 (25) (USA)	Combined GADD45a+PSA+cfDNA: AUC=0.937, SN=94.1%, SP=87.5%
	GSTP1, RASSF1, RASSF2	Real–time PCR	Aykanli et al., 2024 (26)(Turkey)	RASSF2: SN=69%, SP=39%; panel (GSTP1+RASSF1+RASSF2): SP=83%
Plasma	mSHOX2, mSEPT9	MS–qPCR	Krausewitz et al., 2022 (27)(Germany)	mSEPT9: AUC=0.87, SN=75.5%, SP=86.0%; mSHOX2: AUC=0.89, SN=85.3%, SP=84.0%
	RASSF1A, MIR129–2, NRIP3, SOX8	OBBPA–ddPCR	Friedemann et al., 2024 (28) (Germany)	PRISK1: AUC=0.75, SN=100%, SP=52.5%; PRISK2: AUC=0.77, SN=100%, SP=70%
	RASSF1A	ddPCR	Ding et al., 2024 (29) (China)	Cohort 2: AUC=0.874, significantly differentiated PCa from BPH (p=0.03)
Urine	14–gene MDM panel (HES5, ZNF655, ITPRIPL1, SLCO3A1 & others)	TELQAS	Shah et al., 2024 (30) (USA)	SP=100%, SN=71% (overall PCa); SN=59% for clinically significant PCa
	PLA2G16	Pyrosequencing	Jarrard et al., 2019 (31) (USA)	AUC=0.798 vs logPSA AUC=0.519; combined: AUC=0.790
	GSTP1	MS–PCR	Varriale et al., 2019 (32) (Italy)	81.8% of PCa with PSA ≤4 ng/mL had GSTP1 methylation; 0% in healthy subjects
	miR–34b/c, miR–193b (miRNA) + APC, GSTP1, RARβ2 (methylation)	MS–qPCR	Moreira–Barbosa et al., 2018 (19) (Portugal)	Combined: SN=100%, SP=75.0%; Panel 1 (miRNA): SN=95.4%, SP=84.4%
	LGALS3	Pyrosequencing	Abramovic et al., 2024 (33) (Croatia)	AUC=0.664, SN=56.4%, SP=70.4% vs PSA AUC=0.516
Blood & seminal plasma	CAV1	Pyrosequencing	Skara et al., 2023 (34) (Croatia)	CpG1 AUC=0.63, SN=59%, SP=63%; tPSA AUC=0.52

AUC, Area under the ROC curve; SN, Sensitivity; SP, Specificity; MS–qPCR, Methylation–specific quantitative PCR; ddPCR, Droplet digital PCR; TELQAS, Target Enrichment Long–probe Quantitative Amplified Signal; OBBPA–ddPCR, One–bead:one–probe ddPCR; BPH, Benign prostatic hyperplasia; MDM, Methylated DNA marker; cfDNA, Cell–free DNA.

**Table 3:** miRNA biomarkers in liquid biopsy, stratified by sample type.

Sample type	Representative miRNA(s)	Assay method	Study (Country)	Key outcomes (AUC, SN, SP)
Serum	miR–375, miR–141, miR–182, miR–200b	RT–qPCR	Wei Jin et al., 2020 (35) (China)	4–miRNA combination: AUC=0.923
	miR–17–3p, miR–1185–2–3p, miR–4732–5p, miR–1207–3p, miR–4417 (5–cs panel)	RT–qPCR	Liu et al., 2021 (36) (China)	Training: AUC=99.96%, SN=99.3%, SP=100% Validation: AUC= 99.0%
	miR–141	RT–qPCR	Ali et al., 2018 (37) (Egypt)	AUC=0.912, SN=93.3%, SP=80%
	miR–375, miR–26b–5p panel	RT–qPCR	Dülgeroğlu & Eroğlu (38), 2018 (Turkey)	Panel AUC=0.891 (PCa vs non–cancer); AUC=0.944 vs BPH
	miR–26b–5p, miR–98–5p (2–miR model)	ddPCR	Giglio et al., 2021 (39)(Italy)	2–miR model AUC=0.94 (95% CI 0.835–0.954)
	miR–1255b–5p	RT–qPCR	Zhao et al., 2021 (40) (China)	PCa vs BPH: AUC=0.885; Indolent vs Upgrading: AUC=0.909
	miR–4289, miR–326, miR–152–3p, miR–98–5p	RT–qPCR	Matin et al., 2018 (41) (Australia)	Validation: AUC=0.95 (95% CI 0.89–1.00, p < 0.0001)
	let–7b–5p, miR–15a–5p, miR–15b–5p panel	RT–qPCR	Li et al. (42), 2025 (China)	3–miRNA panel: AUC=0.899, SN=85.4%, SP=82.9%
	miR–200c–3p, miR–221–3p, miR–20a–5p panel	RT–qPCR	Wang W. et al., 2025 (43) (China)	Panel AUC=0.880, SN=79.76%, SP=83.33%
	miR–18a–5p, miR–129–1–3p, miR–150–5p, miR–381–3p, miR–106b–5p	RT–qPCR	Chen et al., 2024 (44) (China)	3–miRNA panel: AUC=0.912, SN=91.67%, SP=79.76%
	miR–183, miR–4510, miR–711, miR–329	qPCR	Kumari et al., 2024 (45) (India)	Panel (miR–183+miR–4510): AUC=1.000, SN=100%, SP=100%
Plasma	Exo–miR–21, miR–103, lncRNA GAS5	qPCR + ELISA	Coradduzza et al., 2025 (20) (Italy)	Exo–miR–21 BPH vs PCa: AUC=0.9986; GAS5 BPH vs PL: AUC=0.9556
	miR–106a–5p, miR–148a–3p	RT–qPCR	Coman et al., 2024 (46) (Romania)	Combined: AUC=0.856
	miR–26b–5p, miR–98–5p, let–7a–5p, miR–21–5p, miR–30c–5p	NGS + ddPCR	Giglio et al., 2021 (39) (Italy)	miR–26b–5p: AUC=0.89; 2–miR model: AUC=0.94
	miR–221, miR–141, miR–21	RT–qPCR	Kim et al., 2021 (47) (Korea)	miR–221: AUC=0.98 (vs PSA AUC=0.86)
	miR–106a, miR–130b, miR–223 ratios	RT–qPCR	Sharova et al., 2016 (48) (Italy)	Combined score: SN=83%, SP=81%, AUC=0.84 vs PSA AUC=0.56
	bCaP ratio (miR–375, miR–33a–5p, miR–16–5p, miR–409–3p)	RT–qPCR	Fredsøe et al., 2020 (49) (Denmark)	bCaP+PSA+DRE+age: AUC=0.84
	miR–194–5p, miR–16–5p ratio	RT–qPCR	Temilola et al., 2023 (50) (South Africa)	BPH vs PCa: AUC=0.804; Non– vs metastatic: AUC=0.916
Urine	uCaP ratio: miR–222–3p × miR–24–3p/miR–30c–5p	RT–qPCR	Fredsøe et al., 2018 (51) (Denmark)	C1: AUC=0.95; C2: AUC=0.89; grey–zone (≤15 ng/mL): AUC=0.97
	uCaP ratio (multi–cohort validation)	RT–qPCR	Fredsøe et al., 2019 (52) (Denmark/Spain)	Cohort 1: AUC=0.878 vs PSA 0.741; combined PSA+uCaP: AUC=0.933
	hsv2–miR–H9, hsa–miR–3659	RT–qPCR	Kim et al., 2025 (53) (Korea)	Pivotal grey zone: SN=94.5%, SP=82.7%; miRNA AUC=0.824 vs PSA AUC=0.662
	miR–375, miR–451a, miR–486–3p, miR–486–5p	NGS + RT–qPCR	Li et al., 2021 (54) (China)	4–miRNA panel: AUC=0.979, SN=91%, SP=89%
	miR–335–5p, miR–501–3p ratio	RT–qPCR	Juracek et al., 2023 (55) (Czech Republic)	Best model (ratio+PSA+TPV): AUC=0.7478
	miR–16–5p, miR–195–5p	RT–qPCR	Borkowetz et al., 2020 (56) (Germany)	miR–16–5p/PSAD combination: AUC=0.834, SN=92.3%, SP=87.5%
	miR–100, miR–200b	RT–qPCR	Salido–Guadarrama et al., 2016 (57) (Mexico)	Grey zone PSA: AUC=0.868, SN=67.7%, SP=95.6%
	isomiRs of miR–21, miR–375, miR–204	RNA–seq + RT–qPCR	Koppers–Lalic et al., 2016 (58) (Netherlands)	IsomiRs panel: AUC=0.821; combined+PSA: AUC=0.866, SN=72.9%, SP=88%
	miR–21, miR–214	RT–qPCR	Emamvirdziadeh et al., 2021 (59) (Iran)	Combined panel: AUC=0.721, SN=72.1%, SP=100%
	miR–1913/miR–3659 ratio	RT–qPCR	Byun et al., 2021 (60) (Korea)	Grey zone: AUC=0.821, SN=75.0%, SP=78.6%
	miR–142–3p, miR–142–5p, miR–223–3p	RT–qPCR	Barceló et al., 2019 (61) (Spain)	PSA+miR–142–3p+miR–142–5p: AUC=0.911, SN=91.7%, SP=73.3%
Whole blood	miR–15a, miR–16–1	RT–qPCR	Zidan et al., 2018 (62) (Egypt)	Combined+PSA: AUC=0.91, SN=97.1%, SP=94.3%
	miR–18a	RT–qPCR	Al–Kafaji et al., 2016 (63) (Bahrain)	PCa vs BPH: AUC=0.878
	miR–15a, miR–126, miR–192, miR–377	RT–qPCR	Al–Kafaji et al., 2018 (64) (Bahrain)	Low vs high risk: miR–15a AUC=0.92, miR–126 AUC=0.91
	let–7a, miR–141, miR–145, miR–155, miR–375 panel	RT–qPCR	Kelly et al., 2015 (65) (Ireland)	4–miRNA panel: AUC=0.783, SN=97%
Saliva	hsa–miR–200b, hsa–miR–331–3p	RT–qPCR	Luedemann et al., 2022 (66) (Germany)	miR–200b: AUC=0.663, SN=81%, SP=55%
Serum + Urine	miR–940	RT–qPCR	Rajendiran et al., 2021 (67) (USA)	Serum: AUC=0.75; combined miR–940+PSA: AUC=0.818; csPCa: AUC=0.851
	hsv1–miR–H18, hsv2–miR–H9–5p	Microarray	Yun et al., 2015 (68) (Korea)	Serum combined: AUC=0.925; Urine combined: AUC=0.923 vs PSA AUC=0.701

AUC, Area under the ROC curve; SN, Sensitivity; SP, Specificity; RT–qPCR, Reverse transcription quantitative PCR; NGS, Next–generation sequencing; ddPCR, Droplet digital PCR; BPH, Benign prostatic hyperplasia; PSA, Prostate–specific antigen; csPCa, Clinically significant prostate cancer; uCaP, Urinary cancer of prostate ratio; bCaP, Blood cancer of prostate ratio; PL, Precancerous lesions; lncRNA, Long non–coding RNA; DRE, Digital rectal examination.

**Table 4:** Comparative diagnostic performance of PSA, DNA methylation, and miRNA biomarkers in prostate cancer detection by sample type.

Biomarker type	Sample	Average AUC (Range)	SN/SP Range	Representative
PSA	Serum	0.52–0.79	60–91%/48–89%	Comparator across all studies
DNA Methylation (single gene)	Serum/Plasma	0.51–0.80	31–83%/29–93%	RASSF1A, GSTP1, LGALS3, CAV1
DNA methylation	Urine	0.64 – 0.87	56 – 95%/63 – 100%	RASSF1A (Ding et al., 2024); PLA2G16 (Jarrard et al., 2019); MDM panel (Shah et al., 2024)
DNA methylation + PSA (combined)	Serum/Urine	0.79 – 0.94	82 – 100%/75 –100%	GADD45a+PSA+cfDNA, AUC=0.937 (Reis et al., 2015); PLA2G16+PSA, AUC=0.790 (Jarrard et al., 2019)
miRNA (Single markers)	Serum/Plasma	0.56–0.98	42–100%/39 –100%	miR–221 AUC=0.98 (Kim et al., 2021); miR–141 AUC=0.912 (Ali et al., 2018)
	Whole blood	0.62 – 0.91	80 – 97%/65 – 94%	miR–15a+miR–16–1+PSA AUC=0.91 (Zidan et al., 2018)
	Saliva	0.65 – 0.66	74 – 81%/55 – 58%	miR–200b; miR–331–3p (Luedemann et al., 2022) limited evidence
	Semen	0.72 – 0.91	74 – 81%/55 – 58%	miR–142–3p + miR–142–5p + PSA (Barceló et al., 2019)
miRNA panel	Serum	0.82 – 0.99	85 – 99%/80 – 100%	5–cs panel AUC=99.96 (Liu 2019); 3–miRNA panel AUC=0.912 (Chen et al., 2024)
	Plasma	0.84 – 1.00	73 – 100%/81 – 100%	Exo–miR–21 AUC=0.9986 (Coradduzza et al., 2025); 2–miR model AUC=0.94 (Giglio et al., 2021)
	Urine	0.70–0.979	67–100%/75–100%	uCaP AUC=0.95 (Fredsøe et al., 2018); 4–miRNA panel AUC=0.979 (Li et al., 2021)
Combined Models (miRNA + DNA methylation)	Urine	–	100%/75%	miR–34b/c, miR–193b + APC, GSTP1, RARβ2 (Moreira–Barbosa et al., 2018)
Combined (miRNA + lncRNA)	Plasma	- 0.87 – 1.00	–	miR–21 + GAS5 + Humanin + MOTS–c (Coradduzza et al., 2025)

Values represent ranges derived from eligible studies. ‘–’ indicates insufficient data for range calculation.

AUC, Area under the ROC curve; SN, Sensitivity; SP, Specificity; PSA, Prostate–specific antigen; cfDNA, Cell–free DNA; lncRNA, Long non–coding RNA; uCaP, Urinary cancer of prostate ratio; csPCa, Clinically significant prostate cancer.

### 3.5 Diagnostic significance of epigenetic biomarkers for prostate cancer

The findings of this systematic review highlight the growing importance of epigenetic signatures as potential non–invasive biomarkers for the early detection and diagnosis of PCa. Both DNA methylation and miRNA biomarkers consistently demonstrated diagnostic potential superior to or complementary to conventional PSA testing. However, their performance varied substantially by sample type, biomarker type, and analytical platform. The stratified analyses presented below address this heterogeneity.

### 3.6 DNA methylation biomarkers

Fourteen studies evaluated DNA methylation markers across serum, plasma, urine, and seminal plasma, with targets ranging from individual genes to multi–gene cfDNA panels (**Table 2**). Collectively, these studies support the feasibility of methylation–based detection, though performance varied markedly across matrices and panel compositions. Multi–gene and multi–marker models consistently outperformed single–gene assays, and urine–based strategies offered practical advantages due to non–invasiveness.

#### 3.6.1 Serum–based DNA methylation markers

Serum was the most commonly evaluated sample type across studies evaluating methylation biomarkers, with GSTP1 and RASSF1A being the most frequently evaluated genes. Individually, these genes yielded moderate diagnostic accuracy: with AUCs ranging from 0.51 to 0.78 for GSTP1 and 0.64 to 0.87 for RASSF1A. The substantial variation across cohorts is likely attributable to differences in assay sensitivity, tumour stage distribution, and the comparator used. Notably, the diagnostic performance of these single–gene markers overlapped considerably with that of PSA, which ranged from 0.52 to 0.64 across comparator studies. This overlap raises important concerns regarding their clinical utility as standalone diagnostic tools and underscores the need for multi-marker approaches or integration with existing clinical parameters.

Multi–marker approaches in serum yielded improved diagnostic performance. While HDAC alone showed moderate diagnostic accuracy (AUC = 0.84), its limited specificity (SN = 95%; SP = 65.25%) restricts standalone clinical utility. In contrast, combining GSTP1, HDAC, DNMT3A, and DNMT3B improved discrimination, highlighting the limitations of single–gene markers and the advantage of multi–gene panels (22).

The most diagnostically informative serum model was reported by Reis et al., who integrated GADD45a methylation with PSA and circulating free DNA to achieve AUC = 0.937 (SN = 94.1%; SP = 87.5%), one of the highest reported across methylation studies (25). This finding supports a complementary role for methylation biomarkers alongside PSA, rather than positioning them as competing diagnostic tools.

In contrast, Brait et al. reported modest performance for MCAM (AUC = 0.66) and ERα/ERβ (AUC = 0.50–0.52), indicating minimal added value over PSA when used individually (24).

Overall, serum–based methylation markers demonstrate moderate diagnostic performance as single markers. However, their clinical utility improves substantially when combined into multi–gene panels or integrated with PSA.

#### 3.6.2 Plasma–based DNA methylation markers

In this category, Aykanli et al. assessed GSTP1, RASSF1, and RASSF2 both individually and in combination. Individually, these markers showed poor specificity (SP = 29–39%), which would result in an unacceptably high rate of unnecessary biopsies if applied clinically. Although the three–gene panel improved specificity to 83%, this was accompanied by a marked reduction in sensitivity to 8%, representing a trade–off that renders the model unsuitable for primary screening (26). By contrast, Krausewitz et al. reported strong and balanced performance for mSHOX2 (AUC = 0.89, SN = 85.3%, SP = 84.0%) and mSEPT9 (AUC = 0.87) in a prospective observational study. The use of a prospective design represents a key methodological strength, enhancing the generalisability of these findings (27).

Friedemann et al. further explored multi–gene cfDNA panels in plasma (PRISK1: RASSF1A, MIR129–2, NRIP3, SOX8; PRISK2), achieving 100% sensitivity at specificities of 52.5–70% (AUC = 0.75–0.77) (28). While specificity remains modest, the PSA–independent design of these panels is a notable advantage, as it enables detection that may be missed by conventional PSA-based screening. Nevertheless, external validation in independent prospective cohorts is essential before clinical implementation can be considered.

Overall, the variability in diagnostic performance across studies likely reflects differences in gene selection, patient populations, and analytical platforms. This heterogeneity underscores the difficulty of cross-study comparison and highlights the need for harmonised protocols and standardised validation frameworks.

#### 3.6.3 Urine–based DNA methylation markers

While all studies in this category demonstrated a measurable diagnostic signal, their relative clinical utility differed considerably. The 14–gene MDM panel, assessed by Shah et al. using the TELQAS platform, achieved 100% specificity with 71% overall sensitivity (59% for clinically significant PCa), supporting it as a potential rule–in test (30). However, the reduced sensitivity for clinically significant disease is a key limitation that must be addressed before widespread clinical adoption.

A distinct approach by Ding et al. involved targeting RASSF1A methylation in extracellular vesicle DNA from first–void urine, achieving an AUC of 0.874 in the validation cohort (29). This strategy is particularly notable for enriching tumour–derived material, which may enhance detection sensitivity. Similarly, Jarrard et al. focused on a single–CpG–site analysis of PLA2G16 CG2, yielding an AUC of 0.798, substantially outperforming logPSA alone (AUC = 0.519). However, the combined model showed only marginal improvement (AUC = 0.790), suggesting limited additive value of PSA in this context (31).

The detection of GSTP1 methylation in 81.8% of PCa patients with PSA ≤ 4 ng/mL by Varriale et al. highlights a clinically important finding, particularly given the limitations of PSA within this diagnostic grey zone (32). This finding positions urinary DNA methylation as a promising tool in this setting; however, the absence of a PSA-matched control group limits the strength of this conclusion.

Finally, a multi–modal epigenetic approach integrating miRNA (miR–34b/c, miR–193b) and methylation markers (APC, GSTP1, RARβ2) reported by Moreira–Barbosa et al. achieved 100% sensitivity and 75% specificity (19). While this represents the highest sensitivity observed in this category and supports the value of integrated epigenetic signatures, the moderate specificity indicates a potential risk of false positives and underscores the need for further validation in independent cohorts.

#### 3.6.4 Blood and seminal plasma methylation markers

Studies evaluating methylation markers in these matrices, either combined or as alternative sources, yielded modest but informative results. Abramovic et al. assessed LGALS3 methylation in both blood and seminal plasma cfDNA, reporting an AUC of 0.664 (sensitivity = 56.4%; specificity = 70.4%), which nonetheless exceeded PSA diagnostic performance (AUC = 0.516) in a cohort with clinical biopsy indication (33). Similarly, Skara et al. demonstrated that CAV1 methylation in peripheral blood and semen modestly outperformed PSA (CpG1 AUC = 0.63 vs. tPSA AUC = 0.52) (34).

These findings suggest that seminal plasma may provide biologically distinct methylation profiles not fully captured in blood-based assays, likely reflecting its closer anatomical and physiological relationship to the prostate. However, evidence in this area remains limited to small-cohort studies, and the moderate diagnostic performance observed indicates that substantial optimisation and validation will be required before clinical utility can be established.

#### 3.6.5 Detection methods for DNA methylation

Across methylation studies, the detection methods included MS–qPCR, ddPCR, OBBPA–ddPCR, pyrosequencing, TELQAS, and methylation-sensitive PCR. Methylation-specific quantitative PCR was the most frequently employed technique, largely due to its established sensitivity, quantitative capability, and relative accessibility. In contrast, more advanced approaches such as ddPCR and OBBPA–ddPCR provided higher analytical sensitivity for detecting low-abundance cfDNA, albeit at the cost of increased technical complexity and infrastructure requirements. Pyrosequencing enabled quantitative assessment of methylation at individual CpG sites, offering site-specific resolution.

However, the heterogeneity of analytical platforms across studies introduces substantial methodological variability, complicating direct comparisons of results and limiting cross-study reproducibility. This lack of standardisation underscores the need for harmonised analytical protocols and quality control frameworks to support robust multi-centre validation and eventual clinical translation.

### 3.7 MicroRNA biomarkers

Circulating miRNAs were evaluated across multiple biological matrices, including serum, plasma, urine, whole blood, saliva, and semen matrices (**Table 3**). A consistent finding across different sample types is that miRNA panels outperformed single–marker assays, achieving diagnostic accuracies comparable to or exceeding those of PSA, particularly within the PSA diagnostic grey zone (3–10 ng/mL). Across independent cohorts, the most consistently dysregulated miRNAs included miR–21, miR–141, miR–375, miR–125b, and miR–221 (**Table 5**). The results stratified by sample type are presented below.

**Table 5:** Recurrently dysregulated miRNAs in prostate cancer across different studies.

miRNA(s)	Expression	Ref.
miR–21	Upregulated	(20, 59, 69, 70)
miR–141	Upregulated	(35, 65, 69, 71)
miR–375	Upregulated	(35, 49, 54, 65, 70)
miR–106a and miR–106b	Upregulated	(44, 46, 69)
miR–182 and miR–183	Upregulated	(35, 45, 61, 72)
miR–155	Upregulated	(65)
miR–222 and miR–221	Upregulated	(47, 73, 74)
miR–18a	Upregulated	(44, 63)
miR–125b	Up/Down	(38, 40, 43)
let–7 family (let–7a, let–7b, let–7c)	Downregulated	(38, 42, 65, 75)
miR–15a, miR–15b, miR–16 and miR–16–1	Downregulated	(42, 56, 62)
miR–26b	Downregulated	(38, 39, 76)
miR–30c–5p	Downregulated	(38, 39)
miR–146a and miR–146b	Downregulated	(43, 49, 55, 77)
miR–342–3p	Downregulated	(42, 55, 61)
miR–103 and miR–195	Downregulated	(20, 56)

#### 3.7.1 Serum miRNA studies

Serum was the most commonly used matrix for miRNA detection (reported in 15 studies). Diagnostic performance varied widely, ranging from modest single–marker AUCs of approximately 0.56 (miR–133a–3p; Li et al.) (42) to near–perfect panel performance achieved by multi–marker panels. This variability highlights the limited reliability of individual miRNAs and reinforces the importance of panel composition.

The highest diagnostic accuracy in this category was reported by Liu et al. whose five–miRNA serum panel (miR–17–3p, miR–1185–2–3p, miR–1207–3p, miR–4417, miR–4732–5p) achieved AUC = 99.96%, SN = 99.3%, and SP = 100% in the training set (n = 425), with comparable results in independent test and validation cohorts (36). While these findings are striking, they should be interpreted with caution. Near–perfect diagnostic accuracy, especially in discovery-phase settings, raises concerns about potential overfitting and emphasises the need for validation in multicentre prospective cohorts with more heterogeneous populations.

Similarly, Kumari et al. reported perfect discrimination (AUC = 1.00) using a two–miRNA panel (miR–183 and miR–4510) in an Indian cohort (45). However, the single-centre design limits the generalisability of these findings. In contrast, more moderate but likely more robust performance was observed in other studies, including a three–miRNA panel (miR–18a–5p, miR–129–1–3p, miR–106b–5p; AUC = 0.912) reported by Chen et al. (44), and a four–miRNA panel with an AUC of 0.95 reported by Matin et al. (41), both evaluated in independent cohorts.

Overall, evidence from serum-based studies indicates that well-designed multi–miRNA panels can substantially outperform PSA. However, significant heterogeneity in panel composition, study design, and population characteristics limits direct comparison across studies and precludes meaningful meta-analysis. These findings therefore underscore the need for standardised protocols and large, multicentre prospective validation studies to establish clinically reliable serum miRNA-based diagnostic models.

#### 3.7.2 Plasma miRNA studies

The two–stage design employed by Giglio et al., comprising discovery by next-generation sequencing (NGS) followed by ddPCR validation across three independent cohorts, represents methodological best practice in this category (39). This was further supported by prospective validation, providing greater confidence in its generalisability compared to single-cohort studies. Similarly, Kim et al. reported that miR–221 was highly discriminatory in plasma (AUC = 0.98), with a 33–fold increase in expression in localised PCa relative to BPH and substantially exceeding PSA (AUC = 0.86).

Coradduzza et al. extended the analysis beyond miRNA by simultaneously detecting exosomal miR–21 and miR–103 alongside lncRNA GAS5, Humanin, and MOTS–c in plasma (20). Exo–miR–21 achieved near–perfect discrimination (AUC = 0.99, BPH vs. PCa), while GAS5 alone yielded an AUC of 0.96. The co–detection of non–coding RNA classes from a single liquid biopsy highlights the potential of integrated biomarker approaches and aligns with findings reported by Moreira–Barbosa et al. (19). Additionally, the plasma miRNA ratio approach (miR–106a/miR–130b; AUC = 0.81 vs. PSA AUC = 0.56) developed by Sharova et al. further reinforces the limitations of PSA in distinguishing localised PCa from BPH, a pattern consistently observed across multiple independent plasma-based studies (48).

#### 3.7.3 Urine miRNA studies

The most extensively validated tool in this category is the uCaP ratio (miR–222–3p × miR–24–3p/miR–30c–5p), developed by Fredsøe et al., and validated across five independent cohorts in two separate publications (51, 52). Cohort–level AUC values ranged from 0.878 to 0.95, and the combination of uCaP with PSA consistently exceeded PSA alone (combined AUC = 0.933 vs. PSA AUC = 0.741). Notably, this superiority was maintained within the PSA grey zone (AUC = 0.644 vs. PSA 0.527 in Cohort 4), where PSA is clinically least informative. The multi–cohort validation design, encompassing both Danish and Spanish populations, confers a level of external validity not present in most studies included in this review.

In contrast, the four–miRNA panel (miR–375, miR–451a, miR–486–3p, miR–486–5p), developed by Li et al., achieved an AUC of 0.979 when compared against healthy controls, but performance decreased to 0.726 when evaluated against BPH (54). This distinction is clinically important, as the relevant diagnostic challenge is discrimination between prostate cancer and BPH rather than healthy individuals. The observed performance gap highlights a common methodological limitation: studies benchmarked against healthy controls may overestimate diagnostic accuracy in real-world clinical settings where BPH is the predominant comparator. This limitation was partially addressed by Kim et al., who focused on a biopsy–naive cohort with PSA 3–10 ng/mL in a blinded clinical trial, achieving SN = 94.5% and SP = 82.7%, substantially exceeding PSA alone (AUC = 0.662) within the clinically relevant grey zone (53).

A methodologically important finding by Koppers–Lalic et al. further warrants attention. They demonstrated that isomiR variants of miR–21, miR–375, and miR–204 outperformed their canonical counterparts in urine (AUC = 0.821 vs. 0.661) (58). This suggests that reliance on canonical miRNA sequences in current panels may overlook diagnostically informative molecular variants. If validated in independent cohorts, these findings could have significant implications for the design of future miRNA-based assays, particularly in enhancing their sensitivity and overall diagnostic performance.

#### 3.7.4 Whole blood miRNA studies

Though based on a limited evidence base, findings in this category demonstrate clinically meaningful improvements over PSA alone. Combining miR–15a, miR–16–1, and PSA, Zidan et al. achieved an AUC of 0.900, representing a diagnostically compelling result (62). In contrast, the four–miRNA panel developed by Kelly et al., which did not include PSA, achieved an AUC of 0.783 with a sensitivity of 97%, reflecting high sensitivity but lower specificity (65). Al–Kafaji et al. further demonstrated that miR–15a and miR–126 in whole blood effectively stratified PCa risk, with miR–15a achieving an AUC of 0.92 for distinguishing low-risk from high-risk disease (64).

The recurrence of miR–15a and miR–16 as discriminatory candidates across both serum and whole blood studies suggests that these miRNAs may exhibit relatively consistent expression patterns across different biological matrices. This consistency strengthens their potential for inclusion in platform-agnostic biomarker panels, though further validation in larger, independent cohorts is required to confirm their robustness and clinical utility.

#### 3.7.5 Saliva and semen miRNA studies

In saliva, Luedemann et al. reported modest diagnostic performance for hsa–miR–200b (AUC = 0.663) and hsa–miR–331–3p (AUC = 0.648) in distinguishing PCa from BPH (66). While not clinically sufficient, these findings provide proof–of–concept that tumour–associated miRNAs can be detected in saliva, though validation in larger, multi–centre cohorts is needed.

In seminal plasma, Barcelo et al. reported moderate performance for individual miRNAs (AUC = 0.72–0.74). However, combining PSA with miR–142–3p and miR–142–5p substantially improved accuracy (AUC = 0.911), exceeding PSA alone (61). The biological rationale for this approach is supported by the proximity of seminal plasma to prostatic secretions, which may allow higher concentrations of tumour-derived miRNAs compared to peripheral blood. Nevertheless, as this evidence is derived from a single study, its clinical applicability remains uncertain and requires further validation.

Collectively, these findings suggest that miRNA panels consistently outperform single-marker assays and demonstrate improved diagnostic performance across multiple biological matrices.

#### 3.7.6 Detection methods for miRNA

Across all 51 miRNA studies, RT–qPCR was the predominant detection platform for both discovery and validation phases, owing to its sensitivity, reproducibility, and relative clinical accessibility. Next-generation sequencing (NGS), including Illumina-based RNA and small RNA sequencing, was used for discovery in several studies (39, 60, 78), with RT–qPCR subsequently employed for targeted validation. Microarray-based profiling was used for initial screening in a smaller subset of studies (49, 68). while digital droplet PCR was applied for validation in one high-powered study (39).

The divergence in platforms across discovery and validation phases is not merely a technical detail. Differences in sensitivity thresholds, dynamic quantification ranges, and normalisation strategies vary substantially across platforms. Consequently, AUC values derived from RT–qPCR-based validation studies are not directly comparable to those obtained from NGS-based discovery datasets. This platform heterogeneity represents a significant limitation of the current evidence base and should be explicitly considered in future systematic reviews and meta-analyses of miRNA diagnostics in prostate cancer.

### 3.8 Comparative Diagnostic Performance of Epigenetic Biomarkers with PSA

The diagnostic potential of epigenetic signatures, including DNA methylation and miRNAs, has been consistently benchmarked against PSA across the included studies, and several important conclusions emerge from this comparison. First, single-gene methylation markers offer only modest improvements over PSA in serum and plasma, whereas multi-gene panels and combined models (such as methylation + PSA or methylation + miRNA) achieve substantially higher diagnostic performance, with AUCs often exceeding 0.90. Second, miRNA-based biomarkers demonstrate the most consistent superiority over PSA across biological matrices, particularly within the PSA diagnostic grey zone (4–10 ng/mL), where PSA’s specificity is limited and often leads to a high rate of unnecessary biopsies.

Among cfDNA methylation markers, LGALS3 achieved an AUC of 0.664 (sensitivity = 56.4%, specificity = 70.4%), exceeding PSA alone (AUC = 0.516) in a biopsy-indicated cohort (33). Similarly, PLA2G16 yielded an AUC of 0.798 compared to a PSA AUC of 0.519, representing a meaningful improvement that could translate into reduced unnecessary biopsies if validated in prospective settings (31, 34). Tumour suppressor gene methylation markers, particularly RASSF1A and GSTP1, also consistently outperformed PSA, with reported AUCs ranging from 0.64 to 0.80 compared to approximately 0.60 for PSA alone (21).

In contrast, circulating miRNA-based biomarkers demonstrated the greatest overall improvement over PSA. For example, the miR–21/miR–214 panel achieved an AUC of 0.721 compared to 0.620 for PSA (59), while a four-miRNA urine panel (miR–375, miR–451a, miR–486–3p, miR–486–5p) reached an AUC of 0.979 (54). Similarly, miRNA ratios such as miR–106a/miR–130b/miR–223 achieved an AUC of 0.84, consistently exceeding PSA performance (AUC range: 0.56–0.69) (55) (79) (48),. Notably, integrating PSA with miRNA markers further enhanced diagnostic accuracy, yielding AUCs of 0.84-0.93 across multiple independent studies. Collectively, these findings reinforce the view that epigenetic biomarkers are most clinically valuable as complementary tools to PSA rather than as standalone replacements (**Table 4**).

### 3.9 Recurrently dysregulated miRNAs across different sample types

**Table 5** summarises miRNAs consistently dysregulated across independent cohorts and matrices. Among the most robustly replicated were miR–21 (upregulated in serum, plasma, and urine), miR–141 (upregulated in serum and whole blood), miR–375 (upregulated in serum, plasma, and urine), and the let–7 family (downregulated across serum and plasma studies). The recurrence of these candidates across geographically and technically diverse studies suggests they reflect genuine biological dysregulation in PCa rather than platform–specific artefacts. Notably, miR–26b appeared as a high–performing candidate in both serum and plasma, and miR–15a/miR–16 showed consistent dysregulation across serum and whole blood studies, suggesting potential matrix–independent utility.

## 4 Discussion

The aim of this systematic review was to evaluate the diagnostic potential of epigenetic biomarkers in liquid biopsies for early PCa detection. Across the 67 included studies, epigenetic biomarkers showed high diagnostic performance; however, this must be interpreted cautiously due to the predominance of retrospective case–control designs and methodological heterogeneity. The majority of these results were from retrospective case–control studies with healthy volunteers alone as controls, which does not reflect the clinically relevant diagnostic scenario (24, 37, 44, 59, 69, 80, 81). The relevant comparison is the distinction between PCa and BPH in men with a borderline PSA, where PSA performance drops substantially. The four–miRNA urine panel by Li and colleagues illustrates this plainly: AUC = 0.979 against healthy controls, falling markedly to AUC = 0.726 when compared against BPH (54). Similarly, GSTP1 methylation, often considered cancer–specific, was detected in BPH in several cohorts (32). These findings highlight a critical limitation in current biomarker research: the over–reliance on healthy controls, which inflates diagnostic performance and reduces clinical applicability.

Multi–marker epigenetic panels demonstrate greater diagnostic performance, as they capture multiple independent signals of malignant transformation rather than relying on a single non–specific marker of prostatic disruption (82). This is exemplified by the GADD45a + PSA + cfDNA model, developed by Reis and colleagues, which achieved an AUC of 0.937 (25), while individual components showed lower diagnostic performance. These support the view that diagnostic accuracy improves through complementary biomarker integration rather than isolated effects.

The ConfirmMDx assay represents a successful clinical application of this approach. It is approved in the United States and assesses APC, RASSF1, and GSTP1 methylation in benign biopsy cores to guide repeat biopsy decisions, with a reported negative predictive value of approximately 90% (83). Despite this, many multi-marker strategies fail to progress to clinical use, reflecting the limitations identified in the risk of bias assessment (**Figure 5**), including retrospective designs, non-consecutive sampling, and incomplete reporting.

Similarly, Plasma mSEPT9 is approved for colorectal cancer screening (84), and its combination with mSHOX2 is used for lung cancer detection in Europe (27). In this review, mSEPT9 also demonstrated strong performance (AUC = 0.87) in a prospective PCa cohort (27), suggesting that biomarkers validated in other malignancies may offer a more efficient pathway to clinical translation. Nevertheless, differences in assay platforms, population diversity, and sample types continue to limit cross-study comparability and generalisability.

The detection platforms used across studies introduce important trade–offs that affect both interpretation and translation. RT–qPCR was the most commonly used technique due to its accessibility, sensitivity, and reproducibility. However, it provides relative quantification and is dependent on reference gene selection, as well as sensitive to pre–analytical variables such as extraction methods, haemolysis, and freeze–thaw cycles (85). The AUC range of 0.51–0.78 for GSTP1 methylation and 0.71–0.912 for miR–141 across independent studies in this review reflects both methodological variability and underlying biological variation (22) (21),. Droplet digital PCR offers absolute quantification and superior sensitivity for low–abundance cfDNA targets (39), but its cost and infrastructure demands limit routine use. Similarly, next-generation sequencing enabled essential biomarker discovery, including isomiR variants with enhanced diagnostic value (39, 60, 78), but it is not deployable for routine diagnostics. Notably, no study assessed histone modifications, likely due to technical constraints such as low biomarker abundance and reliance on complex enrichment methods like chromatin immunoprecipitation, rather than a lack of biological relevance (86). Overall, the variability observed reflects both methodological inconsistency and biological heterogeneity, making cross-study comparisons of diagnostic performance difficult. Standardised pre-analytical and analytical protocols, similar in principle to MIQE guidelines, are essential for reproducible multi-centre validation (87). Without such harmonisation, reliable comparison and clinical translation remain limited.

A major finding was also the presence of fewer than 5% of the included studies that involved African populations, despite African men carrying the highest global PCa incidence, disproportionate rates of aggressiveness, and early disease onset (88). This represents a critical, largely underexplored limitation with significant implications for global clinical translation. In addition, epigenetic regulation is highly influenced by environmental exposures, infectious disease burden, and genetics, which differ substantially across populations (89). Consequently, biomarker panels validated predominantly in European and other cohorts may have limited generalisability to African populations. Despite the limited evidence, Temilola and colleagues reported a plasma miRNA ratio with an AUC of 0.916 for distinguishing metastatic from non-metastatic disease in a South African cohort (90), suggesting the existence of population–specific epigenetic signatures. In sub-Saharan Africa, where disease burden is high and laboratory infrastructure is often limited, the implementation of validated, accessible epigenetic biomarkers could offer meaningful clinical benefits (91).

Overall, these findings emphasise the diagnostic potential of epigenetic biomarkers in PCa, while highlighting the urgent need for standardised methodologies and inclusive validation across diverse populations to enable meaningful clinical translation.

## 5 Translational insights and future directions

Epigenetic biomarkers show strong potential to improve PCa diagnosis by enhancing risk stratification and reducing unnecessary biopsies, with feasible implementation across diverse clinical settings due to existing laboratory infrastructure. However, clinical translation is constrained by limited standardisation in sample processing, normalisation, and threshold definition, which affects reproducibility across studies. Evidence from blinded studies in the PSA grey zone (3–10 ng/mL) suggests that urine–based miRNAs may exceed PSA diagnostic performance, though these findings remain insufficiently validated in large, multicentre cohorts.

Clinical translation will require meeting several key requirements. First, validation studies should recruit from the grey–zone population, men with PSA 3–10 ng/mL being considered for biopsy, using BPH as the comparator, with thresholds defined before data collection. Second, performance should be benchmarked against existing risk calculators, such as ERSPC and PCPT, rather than PSA alone. Among current candidates, the uCaP urinary miRNA ratio stands out for its validation across multiple prospective cohorts and its superiority over PSA, suggesting it is closer to clinical applicability than most emerging biomarkers.

## 6 Limitations

With the encouraging findings reported in this review, several limitations are acknowledged. The studies show considerable variation in the study designs, patient populations, sample types, and analytical methods. This heterogeneity precluded direct comparisons and prevented formal meta–analysis. Many studies used retrospective or case–control designs, with small sample sizes, which increases the risk of selection bias and limits the robustness of the conclusions. Reproducibility was further limited by methodological inconsistencies. Differences in sample handling, normalisation strategies, assay calibration, and the definition of diagnostic cut–off values decreased comparability across studies. Additionally, most studies evaluated biomarkers at a single time point, with few comparing changes over time or across disease stages. Importantly, the lack of large, prospective, blinded validation studies remains a major obstacle to clinical adoption.

## 7 Conclusion

Epigenetic biomarkers, particularly DNA methylation patterns and circulating miRNAs, demonstrate strong potential as non–invasive tools for the early detection of prostate cancer. Evidence from this review indicates that these biomarkers are complementary to PSA and, in many cases, outperform it, particularly when used in combination. Future integration of multi–marker epigenetic panels into existing diagnostic frameworks may improve risk stratification, reduce unnecessary biopsies, and support more precise and equitable PCa detection globally.

## Data Availability

The original contributions presented in the study are included in the article/**Supplementary Material**. Further inquiries can be directed to the corresponding author.
